# Pride and pain – a lifespan-informed, identity-affirming approach to paediatric pain in 2SLGBTQIA+ youth: panel discussion at the international symposium for pediatric pain

**DOI:** 10.3389/fpain.2026.1761071

**Published:** 2026-03-18

**Authors:** Jaris Swidrovich, Emre Ilhan, Katelynn E. Boerner, Lauren E. Harrison

**Affiliations:** 1 Leslie Dan Faculty of Pharmacy, University of Toronto, Toronto, ON, Canada; 2 School of Health Sciences and Nursing, Macquarie University, Sydney, NSW, Australia; 3 Department of Pediatrics, University of British Columbia & BC Children’s Hospital Research Institute, Vancouver, BC, Canada; 4 Department of Anesthesiology, Perioperative, and Pain Medicine, Stanford University School of Medicine, Stanford, CA, United States

**Keywords:** 2SLGBTQIA+, adolescent, child, drag artistry, pain, sexual and gender minorities

## Abstract

**Introduction:**

Pain in paediatric populations is increasingly understood as a biopsychosocial phenomenon. However, the experiences of trans and queer youth remain underrepresented in both research, clinical practice, and education.

**Methods:**

This paper is an edited transcription of a panel discussion titled “Pain and Pride” which was hosted by Priss Cryption, a drag persona, at the International Symposium on Pediatric Pain in 2025 at Glasgow, United Kingdom. The aim of the panel was to explore chronic pain in trans and queer youth through an intersectional and lifespan-informed lens. Panelists included Jaris Swidrovich (Priss Cryption), Lauren Harrison, Katelynn Boerner, and Emre Ilhan.

**Results:**

Drawing on lived experience, clinical practice, and research, the panelists were able to highlight the biopsychosocial and political dimensions of pain, the role of identity suppression, and the urgent need for inclusive and affirming care across developmental stages.

**Discussion:**

This paper presented in the form of a dialogue contributes to the growing discourse on identity-informed paediatric pain management and calls for systemic transformation in clinical, research, and educational settings.

## Introduction

Despite growing awareness of diversity in pain experiences (1, 2), 2SLGBTQIA+^1^ individuals remain underrepresented in pain research and underserved in clinical care (3). Across health research more broadly, sexual and gender minority populations are less likely to be intentionally included, adequately measured, or meaningfully analyzed, resulting in substantial gaps in epidemiological knowledge and evidence-based care (4, 5). In pediatric settings, these gaps are compounded by concerns about disclosure, caregiver involvement, and structural stigma, which may further limit visibility and access to affirming pain care (6, 7).

A central contributor to this underrepresentation is the persistent conflation of sex and gender, alongside the routine admission or inadequate assessment of sexual orientation and gender identity (SOGI) in pain research (8, 9). Many studies continue to rely on binary categorizations of male and female without specifying whether these reflect sex assigned at birth, gender identity, or both, thereby obscuring important developmental, social, and biological processes relevant to pain (10, 11). This absence of inclusive SOGI assessment not only limits the interpretability and reproducibility of findings but also reinforces cisgender-heteronormative assumptions that marginalize transgender and queer youth in research and clinical contexts (5, 37). This cis-heteronormativity pervades in how research contextualizes findings of disparities in 2SLGBTQIA+ health outcomes, often pathologizing an individual's identity while failing to acknowledge the systemic and structural factors that create such disparities (12).

Emerging evidence suggests that these omissions are consequential. Sexual and gender minority individuals experience disproportionate exposure to minority stressors, including discrimination, identity concealment, and victimization, which are associate with heightened pain sensitivity, greater pain-related interference, and worse psychosocial outcomes (13–16). Developmental and intersectional frameworks further highlight that pain mechanisms, coping strategies, and clinical outcomes are shaped by interactions between biological processes, socialization, identity development, and structural inequities across the lifespan (17). Together, these findings underscore the need for identity-informed, developmentally grounded, and affirming approaches to pediatric pain research and clinical care. This paper uses an edited panel dialogue to integrate empirical evidence, theory, and lived experience, offering a novel, identity-affirming framework for advancing pediatric pain research and clinical practice.

## Methods

This manuscript presents an edited transcript of a panel discussion titled *Pride and Pain*, at the International Symposium on Pediatric Pain (ISPP) in Glasgow, UK. The aim of this paper is to provide a dynamic and accessible discourse on the clinical management and research on pain in 2SLGBTQIA+ youth. The vibrant, talk-show style workshop—hosted by pharmacist, educator, and drag artist Priss Cryption (Dr. Jaris Swidrovich, Pharmacist, Canada)—brought together three international clinical and research experts: Dr. Katelynn Boerner (Psychologist, Canada), Dr. Lauren Harrison (Psychologist, United States), and Dr. Emre Ilhan (Physiotherapist, Australia). The panel brought a critical and compassionate lens to the study and care of chronic pain in Two-Spirit, lesbian, gay, bisexual, transgender, queer, intersex, and asexual, among other identities (2SLGBTQIA+), youth who are at the intersection of multiple health inequities yet largely absent from pain discourse.

Dr. Boerner introduced the concept that early developmental, hormonal, and socialization processes within broader gendered environments inform the roots of sex and gender differences in pain (17). She discussed getting comfortable with making mistakes and being accountable in an evolving field, and thinking beyond hormones to intersectional social influences in pain.

Dr. Harrison shared emerging empirical data on 2SLGBTQIA+ youth with chronic pain, highlighting disparities in pain interference and psychosocial functioning and examining minority stress as a mechanism driving these inequities. She emphasized the importance of systematic and repeated assessment of sexual orientation and gender identity in both research and clinical practice, and the potential protective roles of social support and community connectedness.

Dr. Ilhan introduced the concept of “reflexion” (with an x) or reflexivity, a relational form of reflection that interrogates power dynamics and positionality in research and care. To address the health inequities impacting 2SLGBTQIA+ youth, researchers and clinicians should actively engage in reflexivity (18) to identify, challenge, and ameliorate these inequities in their practice. Drawing on critical theory, he highlighted how queer joy, gender euphoria, and collective resistance can disrupt oppressive structures that perpetuate inequity, inviting clinicians and researchers to examine their positionality and reflexivity in relation to marginalized communities. Critical theories like intersectionality [e.g., see (19)] for how an intersectional framework can be used to explore chronic pain health inequities) provides a framework for understanding how individuals may be marginalized based on multiple often intersecting ways and that to genuinely ameliorate health inequities require consideration of all forms of societal oppression (e.g., racism, colonialism, homophobia, transphobia, misogyny, sexism, etc.).

Priss Cryption closed the session by illustrating how drag artistry can serve as a radical act of healing and inclusion within pediatric pain care and research—transforming clinical spaces into ones of empowerment. Through dialogue, critical reflection, and audience engagement, the session explored how identity, stigma, and social context shape pain experiences, and how inclusive, affirming approaches can advance both science and pain care.

The transcript presented in the following section was edited from a verbatim version by the authors to improve clarity and consistency of language. While the conversation was fluid, sections of the original conversation have been signposted using subheadings to help capture relevant core themes that emerged during the panel discussion.

### Transcript of panel discussion: pride and pain—navigating chronic pain in 2SLGBTQIA+ youth

*Priss:* Welcome to the first Pain and Pride talk-show—my first time in drag at a professional conference! Priss was created for pediatric pain. We already know that creative modalities—art therapy, music therapy—promote healing. So why not “drag therapy”? It can be healing for the drag artists as well as for trans and queer youth who need role models.

I wish I had an adult for myself to model this. In Saskatchewan, where I'm from, I know there are many children and youth who live with emotional pain of masking themselves all the time. We often talk about how others perceive pain, such as Indigenous Peoples like me who perceive pain as more than physical pain but also mental, emotional, and spiritual pain - but we don't do much about that in our mainstream health systems. As an example, spirituality is a big part of many Indigenous Peoples' health and wellness journeys – but pain clinics and other services for people living with pain generally do not include spirituality. I'm thinking a lot about the pain of hiding your identity and the spiritual toll of consistently pretending to be someone you're not.

When I was a child in Saskatchewan, I wanted an Aladdin and Jasmine doll for my birthday, but all I heard was pushback and interrogation about why I wanted dolls. Moments like this told me that who I was wasn't okay. So, I had to pretend to be someone else in order to attempt to “fit in”. The pain of hiding your identity is profound. Your tone of voice, posture, clothing choices, and more become an act of vigilance. The constant suppression of identity is exhausting and, as many would describe, painful.

For me, for 34 years, I didn't come out and share this about myself. In my opinion, Saskatchewan remains unsafe for 2SLGBTQIA+ people—new laws even require teachers to “out”^2^ students to parents. We're creating all this violence towards trans youth. Creating spaces of safety and representation is vital. I'm a pharmacist and professor, dressed like this, and surrounded by people who are so smart and global leaders in their professions. It's so scary. Very scary. I needed someone to role model this for me but never had it, so here I am to model it for others. My goal? To make sure there's a drag queen at every pediatric pain conference from now on, and all of us working on pain in sexual and gender minority kids.

### Research, representation, and the importance of community

*Lauren:* I'm a cisgender lesbian psychologist currently living and working in the San Francisco area of California. I grew up in Houston and came out to a small and select group of friends at 15, formally coming out to my parents and others at 21. Even after this, it took me awhile to come out professionally and for much of my career I kept my personal and professional identities separate—it felt safer. Working with Katelynn and others helped me integrate the two and expanding my program of research to focus on queer youth with pain has been incredibly rewarding—personally and professionally. Given the current political climate in the U.S., this work is both urgent and risky. When I began exploring pain in sexual and gender minority youth, I realized how little data existed, often due to lack of assessment of these identities in youth. Anecdotally, however, as a clinician in our pediatric pain clinic at Stanford, I had many patients identifying as gender diverse, and once we integrated an expanded assessment of sexual orientation and gender identity into our assessment battery, roughly 26% of patients in our clinic identify as gender-expansive—compared to ∼9% reported in the general population (13). My hope for my research is to examine how minority and structural stressors affects physiological systems in these youth and increase risk for the development of chronic pain and inform the development of affirming pain care.

*Priss:* Student surveys at my university show 25% of students identify as 2SLGBTQ+, up from 10%. I think we're doing better. A lot of folks are rationalizing the numbers. I don't think the proportion of people who are 2SLGBTQ+ has actually changed. I think more people are starting to share it; we've created spaces where people feel more comfortable sharing it. I don't know how much we can rely on the numbers from pediatric pain clinics, though – the youth will likely be under the direct watch of their caregiver adult(s), so they may not share such parts of their identity if they do not feel safe to do so. We have more work to do.

*Lauren***:** Yet funding mechanisms, and even understanding of why this research is important, often lag behind. I once had a program officer at the NIH [National Institute of Health] ask *why* I would hypothesize queer youth would experience disproportionate stress compared to cisgender and heterosexual youth and suggesting that sexual orientation and gender identity were a choice. That mindset underscores why structural change is so critical.

*Priss*: What makes you excited in your research?

*Lauren*: Increasing the representation of the experiences of queer youth with pain makes me excited. And partnering with friends and colleagues to push this forward. Katelynn and I have collaborated over the past couple of years, and it is always exciting and meaningful when people come and talk to us after a presentation. It reassures me that people know this work matters and is important. And that's energizing because, for me, there are parts of this work that is really scary. Two years ago was the first time I spoke about this research on stage at conference, and it was the first time I positioned myself as a queer ciswoman on a professional stage, and today I am sitting on this panel. That excites me. The possibilities for the future excite me.

*Priss*: I applaud you and your bravery. I've been jealous of my colleagues when they give presentations about their work and they don't have to worry “who's going to hate me after this”. We feel such a strong sense of responsibility as trans and queer folks when we dump all of our heart, soul, and personal identities onto the table when we speak.

### Theoretical grounding and reflexivity

*Katelynn*: I'd like to start by positioning myself: I'm a cisgender, straight, white woman, so I want to take a back seat in this conversation—but also use my privilege to help advocate.

*Priss*: How did you go from psychology to sex and gender advocacy?

*Katelynn*: I became interested in sex difference in pain, but I became tired of doing t-tests and saying differences were just due to hormones. It didn't fit with how I understood the world. I turned to feminist scholarship to help me understand how *gender* and *social context* might play a role. But critically, I'm only interested in feminism informing my science if it's trans-inclusive.

[Panelists and audience snap their fingers]^3^

*Priss*: I so admire the work you do and some of your recent publications. For example, I love what you have said about fulsome reporting of study participants. Some studies say 72% of the respondents were women – and that is it. I am left wondering, “Who were the other 28%?” There are many other gender identities, and it is not possible or right to assume the remainder of participants are cisgender males. I would love to see more than “male”, “female” and “other”. What is “other”? There's so much “other” in being seen for what you are and who you are. I identify as two-spirit and male – only one of them can be clicked on forms. Don't limit people's identity.

*Katelynn*: I think a lot of the people are afraid because they don't feel they know how to do it. I've made mistakes—language I used even five years ago makes me cringe – I didn't know better. I've been privileged to work with people who are more informed and I try to prioritize having diverse identities represented on my team. I often feel like I’m not sure if I belong in this space, especially when I make mistakes. To anyone else who may be feeling that way, someone once gave me the amazing advice: “you do the best you do, and then you learn, and then when you know better, do better.” I need to be ready to be called out, so I can learn.

*Emre:* I'm a cis gay man and a physiotherapist. I came out in my mid-twenties. It was scary but liberating. My early research focused on neonates, where “sex assigned at birth” is routinely recorded. Only recently have I started connecting the dots between queerness, the trans experience, and pain in older paediatric populations – an area that has historically been ignored but needs urgent attention.

There's a changing global consciousness about gender, sexuality and pain – people are starting to talk more about pain in 2SLGBTQIA+ youth, and it's only been recent. Like Katelynn, I look back at my papers and think “oh gosh – did I really ask about sex, and why am I thinking in such binary terms”! When I really think about it, I'm alerted to the systemic drivers for these assumptions. There is also a lack of role models in the clinical and research space who are willing to think beyond cis-heteronormative binaries in pain! I hope this is changing, and the discourse in pain care and research is becoming more diverse and inclusive.

*Priss*: I think in spaces like this, we're expected to be the near-perfect superstars in our field, and we have a level of discomfort and fear. I know a lot of people know about reflection, how about reflexion, with an x?

*Emre*: I think of reflection as putting a mirror up to yourself and analyzing your feelings about an event, scenario, etc.: what was good about it, what was bad about it. It's very introspective. Reflexion with an x takes us further than simple introspection—it's relational (18). In a way, it makes us aware of where we are and how we are positioned amongst “it all”. With reflexion, you are able to begin to identify how are you are located amongst others in a situation. For example, reflexion could be done when recruiting a participant for a study, analyzing data, or treating a patient. We could ask questions like: what implications do your social positions have in terms of power dynamics? How do you re-enforce cis-heteronormativity in your practice, and your research? How do we challenge or undo cis-heteronormative assumptions in our practice? For me, reflexivity is a critical skill to have as a health professional, a researcher, and an educator. Reflexivity doesn't get taught enough in clinical and research training!

### Protecting youth and ethical practice

*Priss:* How do you protect the identities of youth who may not be out?

*Lauren:* It's constantly evolving. For one of our qualitative studies (20), we had versions of flyers that were advertising explicitly and more implicitly what the study was focused on (e.g., we are interested in understanding the experiences of LGTBQIA+ youth with pain vs. we are interested in understanding the experiences of youth with pain). QR codes on study fliers allow youth to privately sign-up, and during initial eligibility screening with our team we ask whether they were comfortable having their identity shared with their parents. We also eliminate explicit description of what the study aims are in the consent form to protect youth who are wanting to participate but are not out to their parent. Having queer young adults on our research team helped immensely.

### Politics, pain, and intersectionality

*Priss:* Anti-trans legislation and anti-trans rhetorics create real suffering. What does politics have to do with pain?

*Emre:* As 2SLGBTQIA+ people, being apolitical is not an option. Laws and policies shape our opportunities, safety, and our right to thrive. Most of these laws are colonial, homophobic and transphobic. Pain is biopsychosocial—and therefore it is political by extension. During Australia's marriage equality referendum, the government asked citizens to vote on whether gay people could marry. The ensuing debate inflicted immense stress and stigma towards queer people (21). Imagine hearing your basic rights debated at the dinner table amongst family, in the community, and in the media as a young person—of course that affects the experience of pain, there's no doubt about it! Vilification for being queer or trans causes undue stress and psychosocial harm, and these impact on physical wellbeing and pain!

*Priss*: Everything is scary, and there's a lot of pain and suffering going on, which is very hard to ignore. Everything we say—or don't say—is political. There has been such an anti-trans and anti-queerness push globally. There have been bomb threats called in at drag queen story hour events. For me, looking at the USA election results - it seems like 72 million people at a minimum don't think that I should exist. There's a lot of pain—politics and pain! There are certain realities that trans and queer can't ignore. Like the UK court decision to define a woman based on biological sex!

*Katelynn:* Intersectionality^4^ adds another layer. It's not just a statistical or methodological consideration — it's a way of thinking about systems and structures of power, privilege, and marginalization (24). You might never be able to create a study that takes up all those intersecting identities, but intersectionality can provide a broader way of thinking about how you're asking questions, the social-systemic implications, and where you're moving next.

*Priss*: Every policy and practice we have in this world, we've created it—we can fix it! How about intersections of 2SLGBTQIA+ identities with neurodiverse identities? Where do you see that research as going?

*Katelynn:* Neurodiversity is another crucial intersection. When I was doing my first qualitative study with trans and gender diverse youth (25), a third of our sample said you can't understand our experience without thinking about neurodiversity – and we hadn't even asked about it! Living with a neurodiverse identity that might interfere with their ability to navigate their pain and affirm their gender identity at the same time. For some, chest binding was important but triggered their pain and sensory sensitivities. Individuals are forced to navigate social environments that are based on the dominant neurotype but are already being invalidated because of their gender identity (26). They might think that now that the health professionals know I'm also autistic, I'm definitely not going to get taken seriously. If we want to do service to these young people, understanding these overlapping identities is essential.

*Priss*: You know, cisgender folks have always had access to gender affirming care – wigs, toupées, erectile dysfunction medication, make-up, and more. Why is it a problem when it's a trans person needing gender affirming care? How important do you think it is for a clinician to acknowledge their own sexuality or gender identity as a researcher or doing this kind of work?

*Lauren*: I think back to my younger self—I think of therapists and other clinicians I saw over the years—and if I encountered a provider that was openly gay, I think it would have made a big difference. To see that person as a successful professional, existing…thriving as a queer person would have given me a lot of hope because for a long time, I did not believe that was possible. For me now as a clinician, I always ask myself, what's the function of disclosing my identity to this patient? Is it for me, or for them? If it feels like it is more for me and not necessary or relevant to the care I am delivering, I don't tend to do it. But, if it's to demonstrate that this is a core component of my identity that I am open about and proud of, and it creates representation and space, I always do it.

*Priss:* At the global pain congress last year, I sat through presentations about pain and adverse and positive childhood experiences. We don't name those in pharmacy education. Sitting through those presentations was transformational.

Watching the show *Heartstopper* was so healing, and I think I vicariously achieved so many positive childhood experiences that I didn't get to have in real life. It was just so beautiful how people came out to their teachers, friends and families. I needed that teacher, I needed those friends – I needed friends, period! I think that has a big intersection with pain, as 2SLGBTQIA+ youth, our identity becomes a target for abuse, being bullied. It's a sad story: I never had a birthday party, because I didn't have any friends to invite. I was so jealous of those kids with treat bags. Generally, you keep who you are hidden and you're out on your own. I didn't want my family to feel sad for me. I never shared about the bullying until I was 34. I already had few people and peers in my life, and my fear was that if I share my true identity, I wouldn't have anyone. Every human should be watch that show, *Heartstopper*. It should be a requirement of being a human, regardless of your own identity. You will see something modelled in that show that you can use in your own life.

### Moving beyond biological essentialism

*Priss*: Katelynn - why has pain research focused so much on hormones?

*Katelynn*: Sex hormones is such an obvious explanation for sex differences in pain, especially because sex-based differences in rates of chronic pain and responses to experimental pain become statistically significant around adolescence (27, 28). But there are so many other things going on in the lead-up to and during adolescence. For example, parents respond differently to babies' cries depending on if they're boys or girls (29), and care can be different depending on whether a person is carrying a female or male fetus (30, 31). Certainly, during adolescence you're developing and understanding your own identity and gender, so this should be studied alongside hormones. Assuming that everything is due to biological factors is not telling the whole story: how you are socialized and how you understand that in yourself is just as important. Exploring your gender identity is a normal part of identity development, but society pathologizes it in trans and queer youth (32).

*Priss*: Most of us are set up with a gender, before we're even born. If my parents had held a gender reveal party, boy would they be disappointed. What did having a boy mean? You're having a baby with a penis, that's what that meant! Also, colour doesn't have a gender, and I can only imagine how that child is being socialized, because you’re already thinking of the colour of the wall, and what you think their gender identity should be. Waiting to pain a baby room until the gender is known makes no sense to me.

*Katelynn*: It's so deeply ingrained. I understand some people's reluctance, even if you're thinking of yourself as a progressive person. I catch myself all the time making gendered assumptions and letting that inform my behaviour, it's so ingrained and deeply socialized. I have been having conversations with colleagues about our deep discomfort of acknowledging that we can also sometimes be part of the problem – recognizing and acknowledging that means sitting in a lot of discomfort as you recognize your own biases.

### Clinical implications and advocacy

*Lauren:* We exist, and are conditioned to exist, in a world that is cis-heteronormative. I cannot count the number of times I have said “my partner” to a new colleague and the response is “what is ***his*** name?” As people and professionals, we have to stop making assumptions and recognize the cis-heteronormative conditioning. I’ve worked with clinicians who are uncomfortable asking patients what pronouns they use, and I think it's imperative that they reflect on why that is and actively fight against the discomfort. Every patient interaction should affirm identity and gender identity. It's a light lift for me as a clinician to say, “I’m Dr. Harrison, I use she/her pronouns” but it has the potential to lift a significant burden off of a patient who trying to assess if it is safe to disclose their identity.

*Priss*: Even using bathrooms can be stressful for trans and gender-diverse youth. They just need to pee—without it being a spectacle.

*Katelynn:* Simple language changes matter — “parent/caregiver” instead of “mom or dad,” “siblings” instead of “brothers or sisters.” Environmental factors matter too: Are your gender-neutral bathrooms accessible? In pain rehabilitation, recommending swimming might sound benign — but not if being in a bathing suit increases gender dysphoria (33). It's also about knowing how to support young people with recommendations, it might be a big ask to send kids on to navigate them on their own. It's also helpful to broaden our understanding of what it might mean to have community. We tend to disparage online communities, but online spaces can be vital community lifelines for isolated youth – especially in remote settings (34).

*Audience question*: I wanted to ask about age of asking about sexuality?

*Lauren*: In our clinic, kids 6 years old and up fill in the surveys. We don't have a decision tree about who gets those questionnaires and who doesn't. In the literature, I often see sexual orientation and gender identity assessed in youth 13 years of age and older, but I believe kids have a sense of their identity at a very young age, even if that identity evolves over time. I don't see any harm in asking. Several parents of younger children have asked questions like “why are you asking my 7-year-old about their gender identity?” to which we state it is best practice and standard of care. Our survey has “I don't know what this question means” and “No response” options. Parents and youth who don't hold diverse identities will move on from those questions within seconds and never think twice about it, but for the 11-year-old who is able to disclose their identity, it can mean everything.

*Emre*: I asked a researcher who was doing research on adolescents 15 years of age and older why he hadn't ask any inclusive questions about gender identity and sexuality in his research, and he responded by saying he wasn't allowed to ask about diverse gender identities and sexual orientation, and that the ethics committee wouldn't allow it. His response kind of took me back and made me ask if everyone has this assumption. There is an inherent reluctance to think beyond the cis-heteronormative binary. I attended a few talks in this conference where we still just hear about boys and girls, and there's certainly no conversation about sexuality and its relationship to pain.

*Priss*: Even something as simple as a flight attendant's announcement for “ladies and gentlemen” to please fasten your seatbelts. The majority of the passengers hear they need to fasten their seatbelts – I hear that my Two Spirit identity isn't valid. This happens all the time and every single instance can make a person feel even more different and “othered” than they may already feel.

*Katelynn*: And if you're doing longitudinal research, ask again and again (35, 36).

### Concluding reflections

*Priss:* Everything we've built in this world—we can change. Healing comes through visibility, connection, and community. There's grief in coming out late in life—but also profound joy. I spent 34 years not knowing the freedom of simply existing. That happiness feels miraculous. So do what you can in the spaces you have—because many people are still hiding.

## Discussion

Overall, the workshop integrated scholarship, drag artistry, and lived experience to challenge dominant paradigms in pain science. *Pride and Pain* directly addressed this gap by bringing together international and multi-disciplinary scholars whose work challenges traditional pain models and expands the field's understanding of identity, embodiment, and context. Priss Cryption's hosting of this talk-show panel was critical in garnering collective enthusiasm by audience members, particularly as the discussion was so deeply intertwined with the panelists' personal narratives, allowing for honest discourse.

This work advances pain science by integrating critical theory, developmental frameworks, and empirical data to highlight how structural and interpersonal stigma shape pain experiences in trans and queer youth—and how inclusive assessment and affirming practices can mitigate these harms. By centering trans and queer youth, embracing intersectionality, and challenging systemic norms, we can move toward a truly lifespan-informed, identity-affirming model of care (see Key Takeaways Box 1). As Priss Cryption reminds us, “Everything we have in this world, we’ve created it—and we can fix it.”

Box 1 **Key takeaways.**

• Despite making up a significant proportion of patients with chronic pain attending pediatric pain clinics, there is a lack of data on sexual and gender minority youth.
• Many researchers and clinicians may not understand or appreciate the unique biopsychosocial stressors that contribute to pain in 2SLGBTQIA+ youth. Experiences of stigma, discrimination and public vilification cause distress and harm which contributes to one's pain experience. These need to be explored in research and clinical practice.
Clinical intake forms, assessments, and other data collection tools must include the breadth of gender identities that exist to truly capture the representativeness of patients and research participants.
Applying an intersectional framework when engaging with 2SLGBTQIA+ youth with pain allows are more comprehensive understanding of the person to guide clinical decision making. For example, 2SLGBTQIA+ youth who are neurodivergent need to navigate multiple oppressions when navigating the clinical world which health professionals have a role in actively addressing.
Health professionals and researchers should actively engage in improving their knowledge and skills related to pain in 2SLGBTQIA+ youth.

## Data Availability

The original contributions presented in the study are included in the article/Supplementary Material, further inquiries can be directed to the corresponding author.

## Footnotes

^1^2SLGBTQIA+ is an inclusive acronym which stands for two-spirit, lesbian, gay, bisexual, trans or transgender, queer and/or questioning, intersex, asexual or aromantic, with the + encompassing the multitude of other diverse sexualities and gender identities.

^2^*To out* is a verb which means to disclose a person's minoritised sexual orientation or gender identity publicly without the person's consent.

^3^Priss had various handmade signs to encourage the audience to laugh, applause, gasp, and snap their fingers. Priss explained that snapping your fingers is a common action in the queer community to physically show agreement or affirmation in what the speaker is saying.

^4^Intersectionality is a term that was coined by Kimberlé Crenshaw (22) and extended by others, e.g., Patricia Hill Collins (23), to help understand how different aspects of a person's identity—such as race, gender, class, sexuality, ability, and others—intersect and interact to create unique experiences of discrimination, privilege, and social inequality.
